# Quantifying dynamical spillover in co-evolving multiplex networks

**DOI:** 10.1038/srep15142

**Published:** 2015-10-13

**Authors:** Vikram S. Vijayaraghavan, Pierre-André Noël, Zeev Maoz, Raissa M. D’Souza

**Affiliations:** 1 Complexity Sciences Center, University of California, Davis CA 95616, USA; 2 Department of Physics, University of California, Davis CA 95616, USA; 3 Department of Computer Science, University of California, Davis CA 95616, USA; 4 Department of Political Science, University of California, Davis CA 95616, USA; 5 Interdisciplinary Center, Herzliya, Israel; 6 Department of Mechanical and Aerospace Engineering, University of California, Davis CA 95616, USA; 7 The Santa Fe Institute, Santa Fe NM 87501, USA

## Abstract

Multiplex networks (a system of multiple networks that have different types of links but share a common set of nodes) arise naturally in a wide spectrum of fields. Theoretical studies show that in such multiplex networks, correlated edge dynamics between the layers can have a profound effect on dynamical processes. However, how to extract the correlations from real-world systems is an outstanding challenge. Here we introduce the Multiplex Markov chain to quantify correlations in edge dynamics found in longitudinal data of multiplex networks. By comparing the results obtained from the multiplex perspective to a null model which assumes layers in a network are independent, we can identify real correlations as distinct from simultaneous changes that occur due to random chance. We use this approach on two different data sets: the network of trade and alliances between nation states, and the email and co-commit networks between developers of open source software. We establish the existence of “dynamical spillover” showing the correlated formation (or deletion) of edges of different types as the system evolves. The details of the dynamics over time provide insight into potential causal pathways.

We are increasingly aware that no system lives in isolation. Understanding the nature of a system of interacting networks is a goal that researchers are increasingly focusing their efforts on^1^,^2^,^3^,^4^. One category of such interacting networks are multiplex networks. Multiplex networks are networks that share a common set of nodes that can be linked via different types of edges that comprise the system. Examples of such systems include networks of nation states that have many types of relationships such as trade, alliances, military conflicts etc.; communication networks of individuals who might communicate by email, or over a social networking website; and a transportation network of a city which consists of systems such as road and railway connections. Such multiplex networks can be thought of as layered networks that have the same set of nodes in each layer and links of a particular type are restricted to a single layer (See Fig. 1).

Multiplex networks, also called multirelational networks, were first explored by sociologists as early as 1953^5^,^6^,^7^,^8^. Multirelational networks were also studied by computer scientists within the context of building better community mining methods^9^,^10^. Recently, the interest from the statistical physics and applied math communities in modeling the dynamics and correlations present in multiplex networks has increased dramatically. Discussing the extensive literature that now exists in this field is beyond the scope of our current work, so we refer the reader to the recent review articles by Kivelä *et al.* and Boccaletti *et al.*^11^,^12^. Since these review articles, there have been several additional contributions made on this topic^13^,^14^,^15^,^16^,^17^,^18^.

From a more specific perspective, correlations in multiplex networks are of great interest. Work by Nicosia *et al.* and Kim *et al.* present models for the growth of multiplex networks and explore the impact of metrics such as degree-distribution and degree-degree correlations^19^,^20^,^21^. Correlations present in static snapshots of multiplex networks, such as interdependence of node properties between different layers, correlations in edge overlap, etc., have also been explored^22^,^23^,^24^. More recently, Nicosia *et al.* extend the notion of degree-degree correlations in the context of multiplex networks, measure these correlations in real data, and present models to reproduce empirical observations^25^. The works discussed above explore the presence of correlations in static snapshots, whereas our focus is on correlations in longitudinal evolution.

From a co-evolution perspective, in many multiplex networks representing real world complex systems, the edge dynamics on one layer is often highly correlated with the edge dynamics on other layers. A thorough understanding of these correlations could help us design better network interventions for the control of these systems. For example, data available on Open Source Software, can be thought of as a multiplex network with two layers: first, the communication between developers (such as email) and second, the collaboration between developers (such as committing code to the same file). Understanding correlations that exist between communications and collaborations would help us plan social and/or coding-related events to increase contribution more efficiently. Organizing social events usually involve a lesser cost when compared to a coding event, and help form friendships among developers that might then lead to them collaborating on a project. Similarly, in other multiplex networks, the cost of intervention may be less expensive on one layer but might help in creating the desired effects on another layer where it might be more expensive or harder to intervene.

In this article we present a systematic way, based on Markov chains^26^, to quantify correlations in edge dynamics of multiplex networks using empirical data. As a first step towards quantifying correlations in edge dynamics, we capture how the presence (or absence) of a link between two nodes in one layer influences the dynamics of the edge between the same two nodes in another layer. Our work presents a quantitative way to uncover the co-evolutionary dynamics of edges in different layers which may lead to the emergence of overlap of edges among distinct layers. Such overlaps have been shown to have a significant impact on dynamical processes such as percolation occurring on the network^27^,^28^.

We start with longitudinal data, i.e., time ordered snapshots, of the multiplex network. From this data, we construct a Markov chain that estimates the probability of observing an edge between a pair of nodes on any layer of the multiplex at the current time step, based on the presence/absence of the edge between the same pair of nodes, on all layers, in the previous time step. We call this model the Multiplex Markov chain, the details of which are explained in the Results section. While the Multiplex Markov chain allows us to empirically determine edge transition probabilities, we cannot directly infer if co-occurring edge dynamics between layers are from true correlations that exist between the layers of the multiplex network or due to chance. In order to discern the presence of correlations between layers we construct a null model that treats each layer independently (i.e., one Markov chain for each layer ignoring the presence of other layers). This enables us to compare the value of the transition parameters obtained from the data with that of the null model. We say that there is *dynamical spillover* when the transition parameters obtained from the Multiplex Markov chain are considerably different from those obtained via the null model (see methods section for details). We have made available the Python code to construct the Multiplex Markov chain and the null model on Github^29^.

Further, we illustrate the tool by applying it to two different multiplex data sets: a network of trade and alliances between nation states; and the co-commit and email networks of developers of an open source software. We show that these networks exhibit dynamical spillover, i.e., the edge dynamics on a layer of these multiplex networks are strongly influenced by the presence of edges in another layer. We also identify potential causal pathways that may influence the co-evolution of these multiplex networks. Although we describe how to construct the model for two layers of a multiplex network, the Multiplex Markov chain and the null model straightforwardly generalize to multiplex networks with more layers.

## Results

### Multiplex Markov chain and the null model

In this section we present the Multiplex Markov chain model that quantifies the correlations present in the edge dynamics occurring on two layers of a multiplex network, and the corresponding null model that helps us distinguish non-trivial correlations from those that occur due to randomness. Inferring correlations in dynamics requires the use of temporal data. We take a data-first approach and begin with time ordered snapshots of the multiplex networks. For simplicity let us focus on two layers of the multiplex network, denoted by calligraphic letters (e.g., U and V). The two layers of the network share the same set of nodes between them but each layer only has edges of a particular type. There are no links connecting instances of nodes that are on different layers, i.e., links are restricted to lie on a single layer. Each pair of nodes in this multiplex can be represented using an ordered pair indicating the presence or absence of a link in each layer. We use lowercase Latin letters (e.g., u and v) to represent the absence of a link and uppercase Latin letters (e.g., U and V) to denote its presence. Each pair of nodes can be in one of four states: (i) no edge between the pair of nodes in layer U or layer V, denoted uv, (ii) presence of edge in layer U only, denoted Uv, (iii) presence of an edge only in layer V, denoted uV, (iv) presence of an edge in both layers U and V, denoted UV.

**Figure 2:**
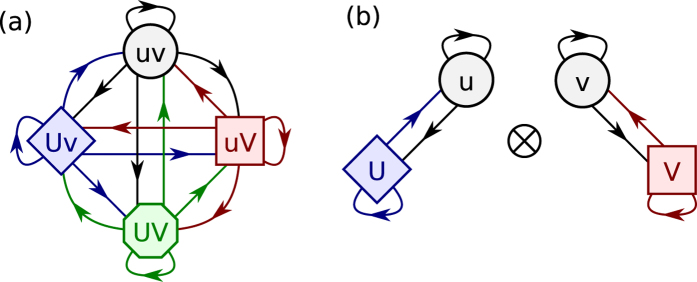
(**a**) Markov chain representation of a multiplex networks with two layers U and V. A lower case letter represents absence of a link in the layer and the upper case letter represents a presence of a link in the layer. Probabilities that are on edges of the same color sum to one. (**b**) Markov chain representation for the edge dynamics on two layers U and V of a multiplex, assuming they are independent of each other. The product of these two Markov chains serves as a null model for estimating the presence of spillover.

We construct a Markov chain describing the dynamics of the edges between a typical pair of nodes in the multiplex network by considering two consecutive snapshots in time, *t* and *t* + 1. Formally, this is what we call the Multiplex Markov chain. Since a pair of nodes can be in one of four possible states in each time step, there are 16 possible transitions in this Multiplex Markov chain. Figure 2(a) shows such a multiplex Markov chain for a network with two layers, U and V. There is a probability associated with each transition that gives the chance of moving to a state at time *t* + 1 given a state at time *t*, which we refer to as the transition parameter. These transition parameters are estimated from the data. Using a Bayesian perspective (see methods) we estimate the underlying probability distribution for these parameters.

As explained above, there are 16 possible transitions (including staying in the same state). Consider every pair of nodes at time *t* and time *t* + 1 and put them in one of 16 possible bins, each corresponding to a transition from a state at time *t* to a state at time *t* + 1. Denote the number of pairs of nodes in each of these bins using 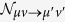 where *μv* and *μ*′*v*′ take values from the set S 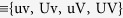. Assuming a uniform prior for the transition parameters, the mean value of each transition parameter, *p*_*μv*→*μ*′*v*′_, can be obtained by normalizing the counts with the total number of transitions leaving from a particular state (see methods). The following expression provides the mean value for the transition parameter based on the data observed


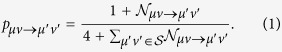


In the absence of any data, the above expression results in each transition parameter having a value of 1/4, thus reflecting our assumption of a uniform prior. One measure of the degree of uncertainty associated with the value obtained above is the standard deviation, *σ*_*μv*→*μ*′*v*′_, of the underlying distribution of the transition parameter. The details of how to obtain these values is described in the methods section.

To distinguish true correlations from sheer chance, we construct a null model to compare with the transition parameters of the Multiplex Markov chain. The null model is defined as a Cartesian product of two Markov chains, one for each layer. This definition ensures that, when the two layers are truly independent, the transition parameters from the null model will be exactly the same as those from the Multiplex Markov chain. We first describe how to construct a Markov chain for a single layer. For a given layer U of a multiplex network, a pair of nodes can exist in one of two possible states: (i) no link exists between the pair, denoted u, and (ii) a link exists between the pair, denoted U. Similar to the Multiplex Markov chain, we can obtain the probability of transition for the single layer from the counts of the transitions 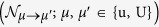 from two consecutive time steps as follows


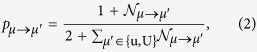


where 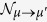 is the number of pairs that go from the state *μ* at time *t* to the state *μ*′ at time *t* + 1. The transition probability of the null model is a product of the transition probabilities for the respective transitions in the single layer Markov chains and can be expressed as


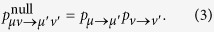


The corresponding spread in the distribution of the transition parameters of the null model can be obtained using the standard way in which parameters involving a product of Gaussian distributions are determined (see the methods section for details).

Having obtained the distribution for the transition parameters of the Multiplex Markov chain and the null model, we can examine the extent to which they overlap. One way to assess such overlaps is to obtain the confidence intervals associated with the estimated parameters. We say that there is positive (negative) dynamical spillover when the confidence interval of the transition parameter of the Multiplex Markov chain lies entirely above (below) the confidence interval of corresponding transition parameter of the null model (see methods section for details; see ref. 29 for code).

### Networks of nations

**Figure 3:**
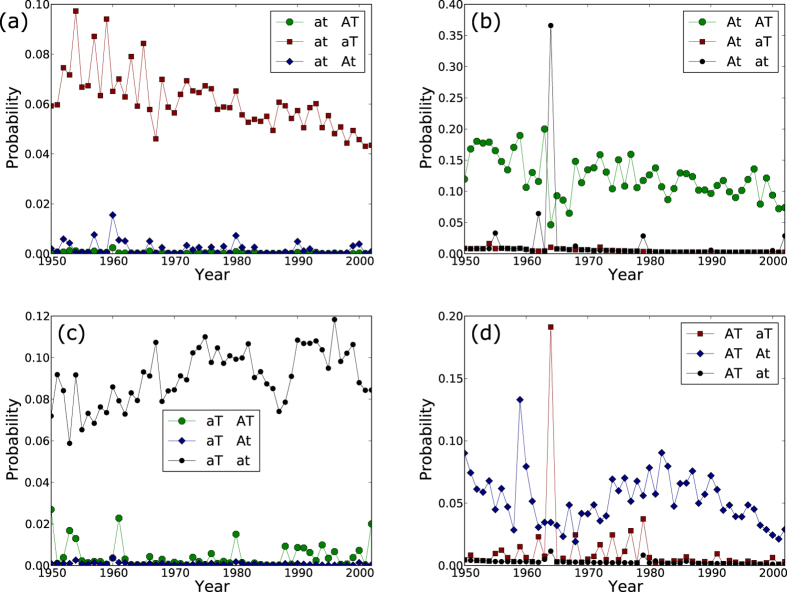
Probability of transition obtained from the Multiplex Markov chain for each year starting from (a) state with no alliance or trade (at), (b) state with only alliance (At), (c) state with only trade (aT) and (d) state with both trade and alliance (AT). The shape of the marker indicates the state to which the transition leads to. Note that the scale of the y-axis is different between the panels.

Different relationships between countries of the world, such as trade, alliances, conflicts, etc., can be represented as a multiplex network with each relationship in a particular layer. A question that is of particular interest to political scientists who study these systems is whether international trade and alliances between nations influence each other. Should there exist a relationship between the two, they seek to understand if such influence is directional in nature. In this section we demonstrate how the Multiplex Markov chain model may be used to analyze such a multiplex network and provide key insights to help answer these questions. Our networks of nations data consist of yearly snapshots of trade and alliances between nations of the world, starting from the year 1950 up to the year 2003. There are 75 nodes (countries) in the year 1950, and this count monotonically increases to 191 in the year 2000. The edges in this multiplex network are alliances (denoted A) and trade (denoted T) which form two layers of this multiplex network. Using the data from each year, we construct the Multiplex Markov chain which results in 16 transition parameters (since there are four states and there are four transitions originating from each of them) for each pair of consecutive years. Figure 3(a–d) shows the time evolution of these transition parameters. We can see that the transition parameters are similar for most years except a few. We perform a principal component analysis (PCA) to examine how similar the transition parameters for the different years are^30^.

The principal component analysis is performed with only 12 of the 16 transition parameters as the features, since the sum of all transition parameters leaving a state is equal to one (and hence not independent). Principal component analysis gives us an ordered list of a linear combination of the 12 features. This list is in the descending order of the amount of variation the linear combinations accounts for. The intent of the PCA is to visualize the structure of the data. In particular, we are interested in identifying the presence of outliers or of distinct clusters. Such structures are highly likely to be apparent in the projection of the data on to the first two principal components. Further, any clusters or outliers not apparent in this projection will, due to the way the principal components are ordered, have a variation that is smaller than that accounted for by the second principal component.

**Figure 4:**
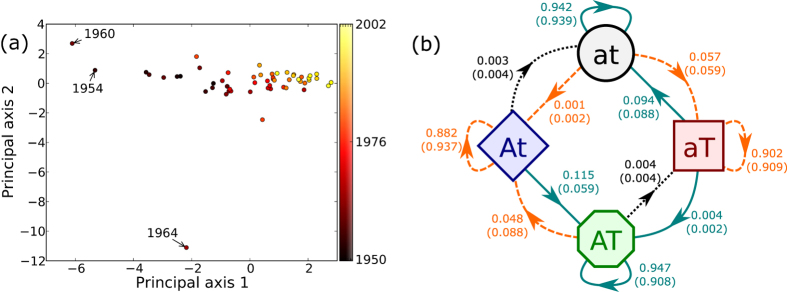
(**a**) PCA of the yearly transition parameters. Note that with the exception of a few outliers most points appear to be similar. The color of the points correspond to the time period (**b**) Multiplex Markov chain obtained by aggregating the transition probabilities for several years. The value of the probability is shown on the arrows with the probability obtained from the null model within parenthesis. Transition parameters having value less than 1 × 10^−3^ are not shown. Solid (dashed) lines represent transitions with positive (negative) dynamical spillover. Dotted lines show transitions without dynamical spillover.

Figure 4(a) shows the two principal component axes, which together explain 55.7% of the variation found in the data. Every point on the figure represents the transition parameters obtained for a given year. The figure shows a trend where the data points at a later time period have a larger value for principal axis 1. The reason is as follows. The major contribution to this principal component axis comes from the transitions At → at, AT → at and AT → aT, all of which have a negative contribution to the first principal component axis. In addition, these transitions, on an average, have a lower value for later time periods when compared to earlier time periods, suggesting that breaking alliance has become less likely over time. Taken together, these two facts help explain the trend observed in the PCA. It is important to note that actual change in the value of these transition parameters between different time periods is smaller than 0.01, but this change is captured and amplified by the PCA because these transition parameters have a large variation in their values. This variation can be seen in Fig. 3(b,d) where these three transitions are close to zero for most years but have a “spiky” behavior for a few years.

Further, we can see that except for the years 1954, 1960 and 1964, most of the years are quite similar and may be aggregated together. Further, we have verified that including these three outlier years slightly changes the numerical values of the parameters but does not affect any aspect of the results discussed below.

The Markov chain obtained by aggregating the counts for the transitions over all the years except 1954, 1960 and 1964 is shown in Fig. 4(b). Note that transition parameters with values less than 1 × 10^−3^ are excluded from the figure because the standard deviation for these parameters are comparable to their mean values. Note that the threshold of 1 × 10^−3^ for the transition parameters is conservative; the value of the largest transition parameter we neglect is 4 × 10^−4^. We first list observations we can make based only on the transition parameters of the Multiplex Markov chain. In all cases, the pairs of nodes in the network have a very high probability of staying in the same state they were present in during the previous year. Consider two nations that do not have a link in either the alliance or trade layer at this moment (at) but end up in a state with a link in both layers (AT) at some point in the future. The Multiplex Markov chain obtained from the data tells us that the states are most likely to first get a link in the trade layer, and then form a link in the alliance layer (in addition to having trade). Also, if two states have an alliance to begin with, they have a very high likelihood of quickly gaining a trading edge in addition to having an alliance.

Having examined the transition parameters obtained from the data, we now focus our attention on dynamical spillover between the two layers by looking for differences between the transition parameters from the Multiplex Markov chain and the null model. We find that, at this aggregate level, the null model does a good job in capturing the probabilities for many transitions. The most notable exception is the transition parameter from At to AT which is much higher when compared to that obtained from the null model. This suggests that the probability that two states establish a trade relationship (with each other) once they have formed an alliance is considerably higher when compared to the two events happening independent of each other. Another difference between the multiplex Markov chain and the null model is the probability of leaving state AT and going back to a state of only alliance (At). This probability is approximately twice as small in the observed data when compared to the null model.

These results shed light on a longstanding debate in the political science literature on the precise nature of the relationship between alliances and international trade. Employing multiple regression models, Mansfield and Bronson^31^ examined the effects of alliances on trade. They found that the interaction between alliance and trade agreements at year *t* increases trade flow between the same countries at year *t* + 1. Additional studies by Gowa^32^, Gowa and Mansfield^33^,^34^, Long^35^, and Long and Leeds^36^, among others, are consistent with this basic finding. Nonetheless, Morrow *et al.*^37^, using a regression model with a different set of independent variables and alternative model specifications, suggest that alliances do not have a robust and consistent effect on trade. From Fig. 4(b), we see that the probability of forming a link in the trade layer when there is no alliance (*p*_at→aT_) is about twice as small when compared to the probability of forming a trade link when an alliance is already present (*p*_At→AT_). We can also see that breaking of a trade link is much more likely when an alliance is absent when compared to when an alliance is present (i.e. *p*_aT→at_ > *p*_AT→At_). These observations are consistent with the adage in political science that “trade follows the flag”, i.e., the forging of economic relations—both during the Cold War era (1950–1990) and the post-Cold War era (1991–2003)—seems to have been affected by the presence of security cooperation between states.

There have also been other studies in political science, such as Fordham^38^ and Maoz^39^, that study the impact trade has on alliances between countries. These studies show that trade increases the likelihood of alliance formation and reduces the probability of alliance dissolution. From Fig. 4(b) we see that the probability of forming an alliance when trade is present (*p*_aT→AT_) is about four times as large as the probability of forming an alliance when no trade is present (*p*_at→At_). This indicates that having trade between two countries helps in the formation of an alliance between them. However, we are unable to say anything conclusive about the effect of trade on the dissolution of alliances. Based on the above results we can see that our method using the Multiplex Markov chain paints a much richer picture and explores potential causal pathways that are found in the data.

### Network of open source software developers

Open Source Software (OSS) projects are great examples of how people come together without a formal organization to collaborate and perform complex tasks. The existence of records of the collaboration activities among developers of OSS projects makes them ideal test-beds for helping uncover the more general question about how cooperation emerges in societies. A problem that has received considerable attention of researchers in this area is the emergence of collaboration among developers and how it is influenced by social ties between them^40^,^41^,^42^,^43^,^44^. Here, we apply the tool developed in the subsection “Multiplex Markov chain and the null model” to a particular OSS project to show how our model may help untangle the relationship between communication and collaboration networks.

**Figure 5:**
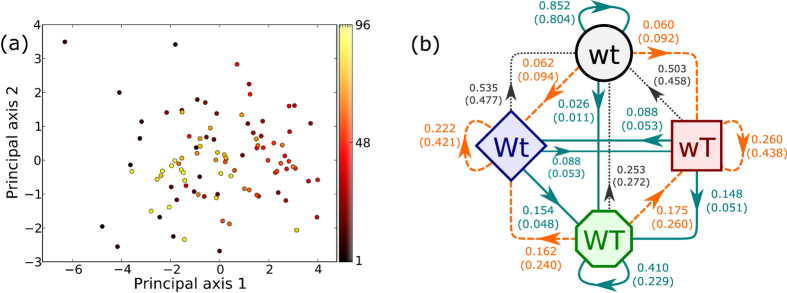
(**a**) PCA of the transition parameters of subsequent 4-week intervals. The color of the points correspond to the time period (**b**) Multiplex Markov chain obtained by aggregating the transition parameters for all 4-week time intervals. The value of the transition parameter is shown on the arrows along with the transition parameter obtained from the null model within parenthesis. Solid (dashed) lines represent transitions with positive (negative) dynamical spillover. Dotted lines show transitions without dynamical spillover.

We study “Apache Axis2/Java”, an open source software project that is part of the Apache software foundation^45^. Our data consists of email communications to a mailing list and co-commits by developers starting from 2^nd^ September 2004 to 19^th^ March 2013. Note that we do not have access to the content of the emails; our data only specifies which developers corresponded with each other. The project had 68 developers who contributed code at some point during our period of observation. The nodes in the network are developers and the two layers are email (T, for talk) and collaboration (W, for work). We use co-commit, i.e., two developers editing the same file, to indicate collaboration between them. We consider a link to be present between two nodes in the respective layer if that activity occurred at least once in a particular four week interval. Therefore our snapshots in time correspond to non-overlapping four week time periods. Different four week time periods have different number of developers. This number ranges between 2 and 29 developers. Due to these number being so small, the data is quite noisy and there is not much we can infer from the raw time series. Therefore, we aggregate the counts for the transitions of the Multiplex Markov chain to discern if there exists dynamical spillover between the email and co-commit networks. Before aggregating, for the same reasons as the analysis of the network of nations, we perform a PCA with 12 of the 16 transition parameters as the features. Figure 5(a) shows the results of the PCA, where the axes shown are the top two in terms of the amount of variation in the data the axes account for. The two axes shown in the figure account for 50.8% of the total variation. There seems to be a single cluster of points and no obvious trend in the PCA. This justifies aggregating the counts for the transitions from the different 4-week intervals.

Figure 5(b) shows the results aggregated over all the time periods. The project has a much higher value for the probability of remaining in the state of having both email and co-commit (WT → WT) when compared to the null, indicating that the correlations between the two layers may play an important role in sustaining collaborations between developers. Moreover, transitions from both Wt and wT states to the state WT exhibit positive dynamical spillover showing that both communication and collaboration between developers can lead to a sustaining state where developers both collaborate and communicate with each other. Studies by Xuan *et al.*^43^,^44^, on open source software using different techniques, agree qualitatively with the results we obtain.

## Discussion

In this article we construct a model based on Markov chains to ascertain the existence of dynamical spillover in real world multiplex networks. By constructing an appropriate null model, we establish how to distinguish the correlations that arise from sheer randomness and those that arise from non-trivial dynamics present in the system; we refer to the non-random correlations as dynamical spillover. In addition, we apply the tool to two real world datasets: (i) trade and alliance between nation-states, and (ii) email and co-commit network of an open source software project. We show that these networks exhibit non-trivial co-evolution among the layers of their respective multiplex networks, and tease out some potential causal pathways.

While the Multiplex Markov chain presented here can be generalized to more than two layers, it is important to note that, for a multiplex network with 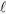 layers, the number of states would be 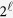, with 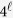 transition parameters. Therefore, when there are a large number of layers, we might need additional assumptions that can decrease the computational complexity of the model. Another consideration is the assumptions about the prior. Here we assume a uniform prior for our Multiplex Markov chain model because it is a good starting point. There can be situations where one might use a prior derived from other types of information, especially when the data on edge dynamics is very sparse. The issue of time scale is also worth discussing: in its current form the Multiplex Markov chain can only detect correlations that are found between two subsequent time steps. Some relationships may depend on the duration for which they have been active. For example, it might be easier to sustain trade between two countries that have already been trading for several years. These types of effects could be captured by expanding the model to include more states that encode memory. In cases where the characteristic time scale is explicitly known, we could construct the time snapshots so that they take this into account thereby enabling the Multiplex Markov chain to capture these correlations. The approach described in this article studies the presence of correlations at the edge level (aggregated over all pairs). A question that future works may focus on is how these correlations affect network structures such as the presence of a core-periphery structure or communities. Future studies could also extend this technique to include neighborhood of the edges and systematically study the effect of such correlations on the dynamics on the network.

## Methods

### Uncertainty in transition parameters of a Markov chain

Both the Multiplex Markov chain and the null model require that we estimate the transition parameters of a Markov chain. First consider a transition parameter *p*_*μ*→*μ*′_ for a single layer. Using the prior that the parameter is uniformly taken from the interval [0, 1], a full Bayesian inference would update on the information 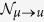 and 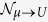 to yield a beta distribution^46^. The mean of this distribution is the value given in Eq. (2). The standard deviation of this beta distribution is a measure of variance in the estimated transition parameter. The standard deviation is obtained using the formula


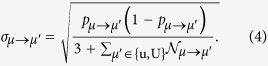


When two layers are involved in the transition, a full Bayesian inference with a uniform prior would yield a Dirichlet (i.e., multivariate beta) distribution of order 4 with the mean value of the different variates given by Eq. (1). The standard deviation associated with the variates of such a Dirichlet distribution of order 4 is given by


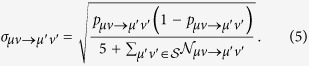


Note that our approach does not need the values for the covariances.

### Uncertainty of the null model

The spread in the distribution of the transition parameter of the null model will depend on the spread in the distribution of transition parameters of the single layer Markov chains from which we construct our null model. Once we have determined the standard deviation of the transition parameters of the single layer Markov chains, we can use them to estimate the spread in the transition parameters of the null model. Since the transition parameters are sufficiently far away from 0 and 1, and since the counts from which we estimate the transition parameters are sufficiently high (of the order of 10^3^), the beta distribution can be approximated as a Gaussian distribution. This leads to the following expression for standard deviation of the transition parameters of the null model.


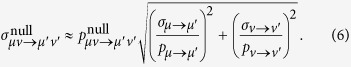


In obtaining the above approximation we have assumed that the term involving the product *σ*_*μ*→*μ*′_ *σ*_*v*→*v*′_ is small when compared to other terms present. Our assumption is justified since 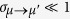 and 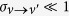 so their product is even smaller. In cases where the data is not sufficient to justify the assumptions made above we can compute the standard deviation by numerically sampling the two beta distributions associated with the null model.

### Dynamical spillover

We say that dynamical spillover exists between layers of a multiplex network if at least one of the transition parameters of the Multiplex Markov chain is “substantially” different from that of the null model. We can say that the difference is substantial when the confidence intervals associated with the transition parameter of the Multiplex Markov chain and corresponding transition parameter of the null model do not overlap. Further, when the confidence interval of a transition parameter for the Multiplex Markov chain lies entirely above (below) the confidence interval of the corresponding transition parameter of the null model we refer to it as positive (negative) dynamical spillover. In this work, we choose the 99% confidence interval for both the Multiplex Markov chain and the null model. Note that the transition probabilities from the Multiplex Markov chain and the null model are positively correlated and hence this is a conservative estimate of whether the transition parameter obtained from the Multiplex Markov chain and null model are indeed considerably different. Python code to discern the existence of dynamical spillover can be found online^29^.

### Data

The data on international alliances is derived from the ATOP project^47^. Trade networks are derived from a combination of two datasets on international trade: the Correlates of War (COW) bilateral trade dataset^48^, and the Gledistch trade dataset^49^. These datasets are combined to reduce the amount of missing data, but they are similar in all other respects. The trade data is binarized such that an edge exists if the value of trade between two countries is greater than 10^−3^ of the exporter’s GDP. Note the above threshold is not symmetric between the two countries involved in trade, and hence gives us a directed network of trade between nations. Neglecting the edge directionality (i.e., ensuring there is at least one-way trade) yields the undirected networks studied herein.

The email network of OSS developers is constructed by scrapping online records of the mailing lists^50^. The co-commit network is obtained as a projection of the bipartite network of developers and the files they commit to. Each commit represents an edge in this bipartite network. The information is publicly available on the git repository of the OSS project. We consider snapshots in time aggregated over non-overlapping four week intervals. Unlike the network of nations, where we know if a country exists in a given year independent of the trade and alliance relationships, in this data we are not sure if a developer is actively contributing to the project and have to infer the existence of the developer based on their activity either in the email or co-commit network. We consider the developer to be part of the system if she/he has at least one edge (in either layer) in the current time step or the next time step. This tends to underestimate the probability of a developer who is neither actively committing code nor sending emails to the mailing list will remain in the same state.

## Additional Information

**How to cite this article**: Vijayaraghavan, V. S. *et al.* Quantifying dynamical spillover in co-evolving multiplex networks. *Sci. Rep.* **5**, 15142; doi: 10.1038/srep15142 (2015).

## Figures and Tables

**Figure 1:**
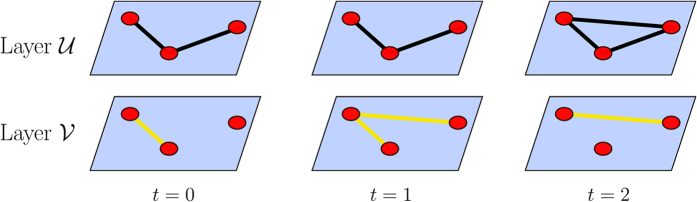
Three consecutive snapshots in time of an abstract multiplex network consisting of two layers, 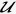 and 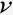. A multiplex network is a network where different layers share the same set of nodes and edges on each layer represent a different relationship. For example, here the nodes could represent countries in the world, the edges in layer U the alliances between them, and the edges in layer 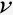 may denote trade between the same set of countries.
